# Low coverage sequencing for repetitive DNA analysis in *Passiflora edulis* Sims: citogenomic characterization of transposable elements and satellite DNA

**DOI:** 10.1186/s12864-019-5576-6

**Published:** 2019-04-02

**Authors:** Vanessa Carvalho Cayres Pamponét, Margarete Magalhães Souza, Gonçalo Santos Silva, Fabienne Micheli, Cláusio Antônio Ferreira de Melo, Sarah Gomes de Oliveira, Eduardo Almeida Costa, Ronan Xavier Corrêa

**Affiliations:** 1 Departamento de Ciências Biológicas, Universidade Estadual de Santa Cruz (UESC), km 16, Salobrinho, Ilhéus, Bahia CEP 45662-900 Brazil; 2 CIRAD, UMR AGAP, F-34398 Montpellier, France; 3 Departamento de Botânica, Instituto de Biociências, Universidade de São Paulo (USP), Rua do Matão, 14 – Butantã, São Paulo, SP CEP 05508-090 Brazil; 4 Núcleo de Biologia Computacional e Gestão de Informações Biotecnológicas (NBCGIB), Universidade Estadual de Santa Cruz (UESC), km 16, Salobrinho, Ilhéus, Bahia CEP 45662-900 Brazil

**Keywords:** Chromosome mapping, LTR retrotransposons, RepeatExplorer, *in tandem* repetitive DNA, Molecular cytogenetics, FISH

## Abstract

**Background:**

The cytogenomic study of repetitive regions is fundamental for the understanding of morphofunctional mechanisms and genome evolution. *Passiflora edulis* a species of relevant agronomic value, this work had its genome sequenced by next generation sequencing and bioinformatics analysis performed by RepeatExplorer pipeline. The clusters allowed the identification and characterization of repetitive elements (predominant contributors to most plant genomes). The aim of this study was to identify, characterize and map the repetitive DNA of *P. edulis*, providing important cytogenomic markers, especially sequences associated with the centromere.

**Results:**

Three clusters of satellite DNAs (69, 118 and 207) and seven clusters of Long Terminal Repeat (LTR) retrotransposons of the superfamilies Ty1/Copy and Ty3/Gypsy and families Angela, Athila, Chromovirus and Maximus-Sire (6, 11, 36, 43, 86, 94 and 135) were characterized and analyzed. The chromosome mapping of satellite DNAs showed two hybridization sites co-located in the 5S rDNA region (PeSat_1), subterminal hybridizations (PeSat_3) and hybridization in four sites, co-located in the 45S rDNA region (PeSat_2). Most of the retroelements hybridizations showed signals scattered in the chromosomes, diverging in abundance, and only the cluster 6 presented pericentromeric regions marking. No satellite DNAs and retroelement associated with centromere was observed.

**Conclusion:**

*P. edulis* has a highly repetitive genome, with the predominance of Ty3/Gypsy LTR retrotransposon. The satellite DNAs and LTR retrotransposon characterized are promising markers for investigation of the evolutionary patterns and genetic distinction of species and hybrids of *Passiflora*.

## Background

The *Passiflora edulis* Sims species (Passifloraceae A. L. de Jussieu ex Kunth), also known as sour passion fruit, is original of tropical climate. Brazil is considered one of the most important centers for genetic diversity of *Passiflora* L., with over than 137 indigenous and approximately 85 endemic species [1–3]. Passion fruit cultures have a high agronomic value due to the production of *in natura* fruit and juices consumption, which reflects the potential consumer market [4, 5]. The last survey for agricultural production realized in 2015 by the Brazilian Institute of Geography and Statistics (IBGE) with reference to the period between 2007 and 2013, placed Brazil as the world’s largest producer and consumer of passion fruit, and the state of Bahia as the largest producer in the country [6]. In addition to Brazil being the main consumer market, the sour passion fruit exportation is booming, representing an important generator of foreign exchange [4, 5].

Plant genomes have a substantial portion of repetitive DNA sequences, which can represent more than 80% of the genome size in certain plant species, as observed in wheat (*Triticum aestivum* L.) and maize (*Zea mays* L.) [7, 8]. Repetitive DNA can be classified by its structure and location, including: (***i***) *in tandem* repeats or satellite DNA (SatDNA) and (***ii***) scattered sequences (transposable elements-TEs) [9–11]. *In tandem* repeated DNAs represent the main component of heterochromatic regions, and can be associated with specific functions in the chromosome, such as centromeres and telomeres. They can be classified based on the repeated unit (monomer), and cluster repetition sizes. Considering the differences in the size of the repeating units, they are classified as microsatellites (1 to 5 base pairs - pb), minisatellites (up to 100 pb) and satellites (hundreds to thousands of pb). Micro- and minisatellites can be found throughout the genome (rarely associated to gene regions) and are the main components of telomeres; while satellites are more frequent observed in centromere and subtelomere regions [12]. Despite the many studies focusing on SatDNA, little is known about their functions [8], in contrast to studies involving ribosomal DNA (rDNA), which consist of *in tandem* moderated repeats, and represent sequences preserved between species and have well-defined functions [13].

Since their discovery by McClintock in 1948 [14], TEs have been subject of many studies and new knowledge continues to be generated. It is currently known that TEs can represent 80% of genomic DNA in plants; as observed in wheat, where TEs represent 68% of the genome [7]. TEs are classified based on enzymology, structural similarities and sequence relationships [15, 16]. In eukaryotic genomes the TEs belongs to two types: a) Retrotransposons, which move in the genome by a reverse transcription of an RNA intermediate, producing a new copy in every replication cycle; and b) Transposons, DNAs that move directly within the genome by a mechanism called “cut-and-paste” [16]. Retrotransposons can be divided into two large groups: Long Terminal Repeats (LTR) retrotransposons, and non-LTR retrotransposons [17]. In general, elements with LTR are more abundant in plants, and elements without LTR and are more frequent in mammals [18, 19]. Our study focused on LTR-retrotransposon, which is characterized by an internal region formed of *gag* genes (*group-specific antigen*, encoding structural proteins similar to the viral capsid), *pol* genes (*polyprotein*, encoding the catalytic protein integrase (INT)), reverse transcriptase (RT) and RNAseH (RH, required for replication), and long terminal repeats [20]. The Ty1/Copy superfamily is the most abundant LTR retrotransposons within plant genomes, differing in the preserved domain of RT genes and in the position of the INT domain within the *pol* complex [21].

Regarding to their chromosome position, the retrotransposons may be present in every chromosome, also observed in centromeric and pericentromeric regions [22]. Retrotransposons associated with the centromere have been found in wheat [23], rice [24] and sugarcane [25]. Mobility and amplification of retrotransposons continuously generate mutations, therefore a source of genome diversity, besides acting in genetic regulation [26, 27]. Thus, the repetitive DNA sequences that are so abundant in plants can be responsible for their genome variation, which may influence the evolutionary distances between species [8, 28]. Centromere region (pCEN38) probes were used in evolutionary studies in sorghum (*Sorghum bicolor* (L.) Moench), demonstrating that sorghum and sugarcane share the same ancestor [29]. In the microalga *Tisochrysis lutea*, families found of mobile TEs were among the three most expressed genes detected in a transcriptional study, important for evolutionary study in microalgae [30].

The amount of sequences of *Passiflora edulis* deposited in public databases is relatively small, limiting the knowledge about its genome [31]. Genomic studies of agricultural crops such as passion fruit are needed to guide the gene manipulation, and can help breeding programs to improve their quality and productivity [32]. Expanding the *Passiflora* genomic studies is important to enhance the knowledge about the structure, function and regulation of the genome, helping the understanding of evolutionary, physiological and morphological aspects.

The Molecular Cytogenetics studies, through the Fluorescent In Situ Hybridization (FISH) technique have allowed the localization of genes and repetitive DNA sequences, allowing the detailed investigation of chromosomal structure [33, 34]. This tool has led to important advances on plant cytogenetics, as well as the verification of the genetic stability during cross-breeding processes, the genitors selection (by selecting plants containing genetic alterations associated with the characteristics of interest), and the monitoring of the amount of genetic material generated by interspecific crossings [35]. In addition, FISH also contribute to analyses of intergenomic pairing in hybrids, and the use of probes containing repetitive elements to detect heterochromatic regions or large number of repetitions that are particularly useful for mapping and evolutionary studies in plant genomes [36].

The construction of linkage maps in *Passiflora* [37, 38] will allow gene sequences of agronomic importance to be located on chromosomes using FISH, integrating cytogenetic and genetic maps, as performed in *Lotus japonicus* L. [39]. Chromosome rearrangements can be identified by changing the location of chromosomal regions, such as centromeric regions. The effects of chromosomal rearrangements can be beneficial, and may result either in characteristics of interest, or can lead to undesirable characteristics, such as plant sterility [40]. The identification and cytogenomic characterization of repetitive DNA in sour passion fruit using FISH may enable the analysis of genomic changes in plants. These sequences might be used as cytogenomic markers to analyze karyotype alterations originated from *loci* movement. These repositioning are often observed in centromeric regions, as verified in evolutionary studies with cucumbers and melons, and in similar species belonging to the genus *Cucumis* L. [41].

This study aims to identify and characterize repetitive sequences in *Passiflora edulis* genome, using Next-Generation Sequencing (NGS) data and bioinformatics analysis by RepeatExplorer [42], and finally produce repetitive DNA probes for chromosome mapping through FISH. Our work focused on the analysis of TEs and SatDNAs associated to centromeres, considering that they are species-specific markers widely used for the identification of chromosomal alterations, an important tool for genetic improvement programs and evolutionary studies of passion fruit.

## Results

### Graph-based identification of repetitive DNA, classification and chromosomal mapping of satellite DNA

**Fig. 1:**
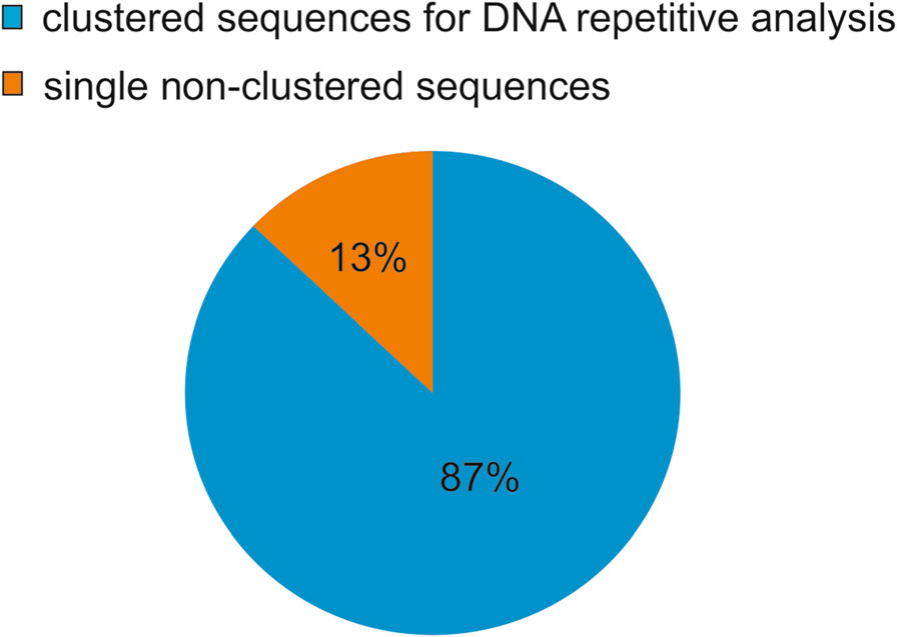
Proportion of total 2,368,626 reads in the genome of *Passiflora edulis* by RepeatExplorer. 87% of the reads were grouped for repetitive DNA classification (blue) and 13% of reads as single copies (orange)

**Fig. 2:**
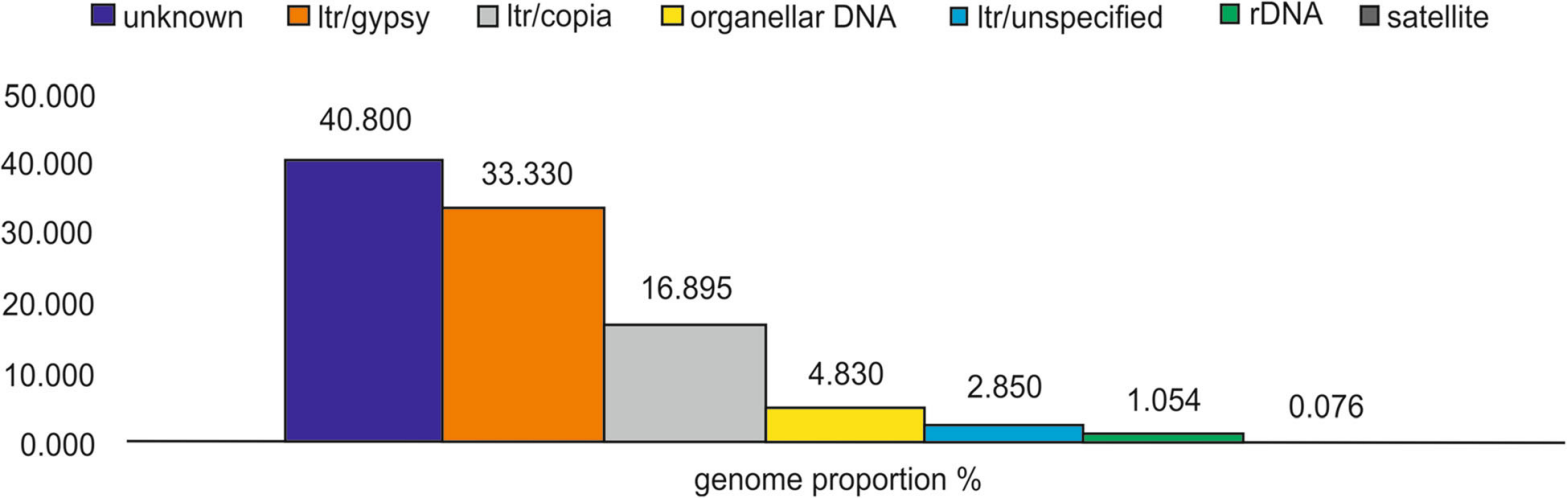
Automatic classification of the most representative clusters (CLs) in the genome of *Passiflora edulis*. The proportion of each CL (%) is shown in the columns

Among the total of 11,493,782 paired-end reads obtained by sequencing using Illumina MiSeq® platform, 2,368,626 reads were analyzed by RepeatExplorer [42, 43]. The paired-end reads were clustered based on similarities and analyzed using graphical representation. The RepeatExplorer grouped 2,059,943 reads as repetitive DNA (87%), and 308,692 as unique, non-grouped sequences (13%) (Fig. 1). Clustering based on reads similarity generated 65,578 CLs. However, 223 CLs have been identified as the most representative (more than 0.01% of reads), containing repetitive elements that are more abundant in the genome. Automatic sorting of the CLs, based on reads homology with databases, enabled the observation of higher proportions of LTR retrotransposons in the genome, totaling 53% of *P. edulis* genome. Ty3/Gypsy superfamily was the most abundant (33.33%), followed by Ty1/Copy (16.89%) (Fig. 2). The reads with homology for rDNA (5S and 45S) had around 1% genome proportion, and the lowest proportion observed was for SatDNAs, reaching less than 0.1% (Fig. 2).

**Fig. 3:**
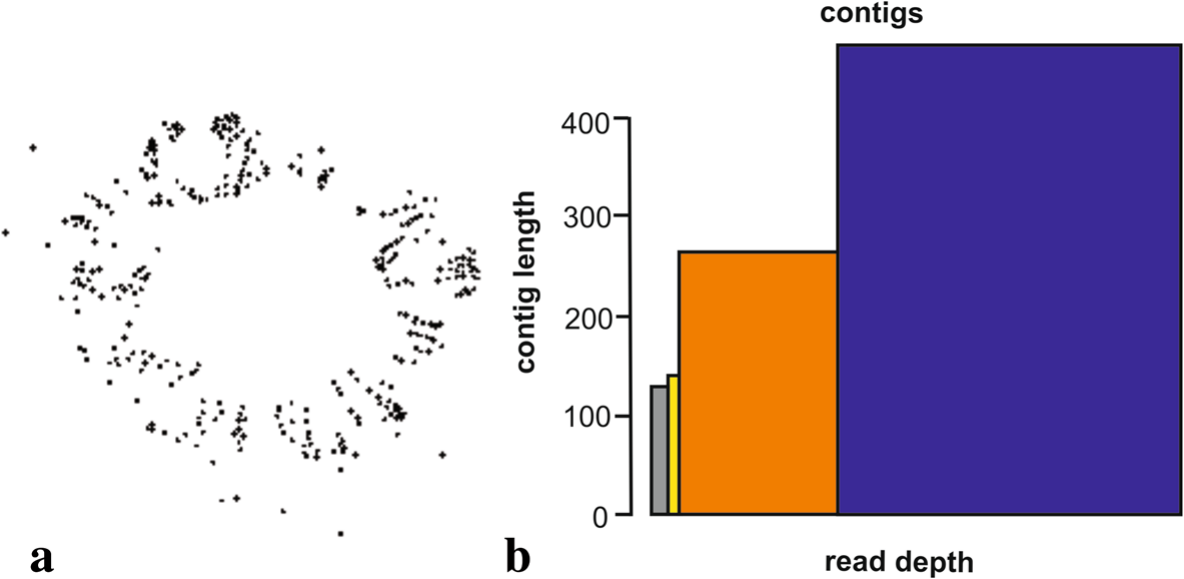
Graphic analysis of CL 207 in *Passiflora edulis*. Graphic layout detected on graph-based clustering analysis (**a**); Contigs distribution according to size and depth of the reads. Contig 1 (blue), contig 2 (grey), contig 3 (orange) and contig 4 (yellow) (**b**)

**Fig. 4:**
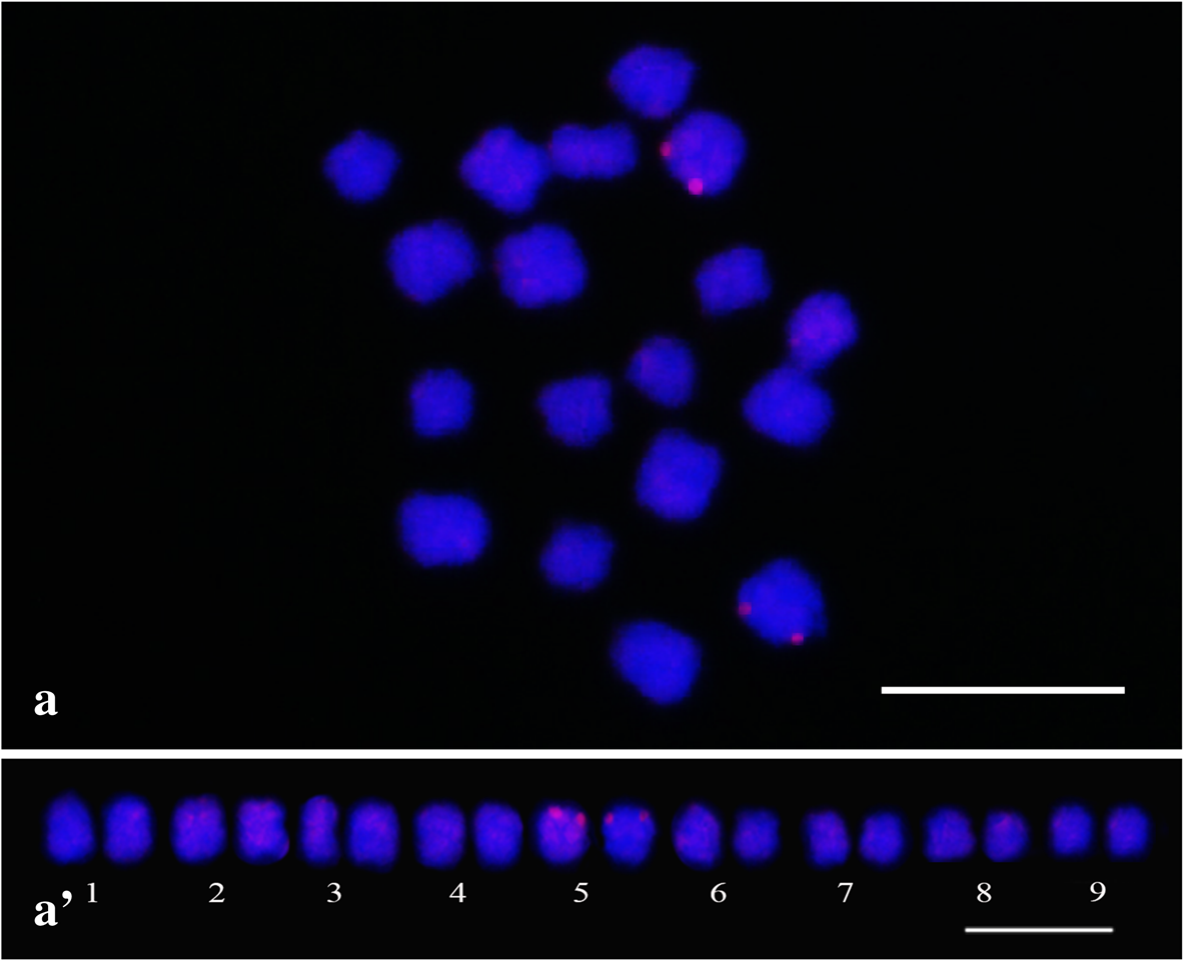
FISH in *Passiflora edulis* (2*n* = 18). Hybridization sites for CL 207 (PeSat_1) (a); karyogram showing signals on terminal regions of the short arms of the fifth homologous pair (a’) (Bar = 10 μm)

The analysis for repetitive elements identification prioritized the elements possibly associated with the centromeric region. The parameters were based on the graphic layout (circular or star-shaped) and homology classification of reads (hits) with the RepeatMasker databases and the customized library (satDNAs and TEs associated with centromere). In the automatic classification, among the 223 CLs, only one was significantly classified as satellite, CL 207 presented the expected patterns for Satellite DNA: graphic showing a circular layout (Fig. 3a), reads homology in the RepeatMasker databank with relevant similarity to satellite (42.45% hits), and 28.85% similarity (hits) to centromeric DNA of *Arabidopsis thaliana* L. from the customized library. CL 207 was composed by only four contigs, which were submitted to BLAST (Basic Local Alignment Search Toll) in the GenBank/NCBI, showing high similarity with the 5S ribosomal gene described for *Beta vulgaris* L. and *Allium tuberosum* Rottler ex Spreng (both with 97% identity, for contigs 1 and 3). The other two contigs (2 and 4) showed no similarities in the GenBank. The initial goal was to locate only the SatDNAs associated with the centromere, for which reason contig 2 (PeSat_1) was selected for analysis and chromosome mapping for presenting greater representation in the genome (depth of reads x size) in comparison to contig 4 (Fig. 3b). The results of FISH revealed two evident hybridization sites at the terminal region of the fifth homologous pair (Fig. 4). The markings were reproducible and unambiguous for all analyzed mitotic metaphases analyzed.

### Satellite DNA identification by tandem repeat analyzer (TAREAN) and chromosome mapping

**Fig. 5:**
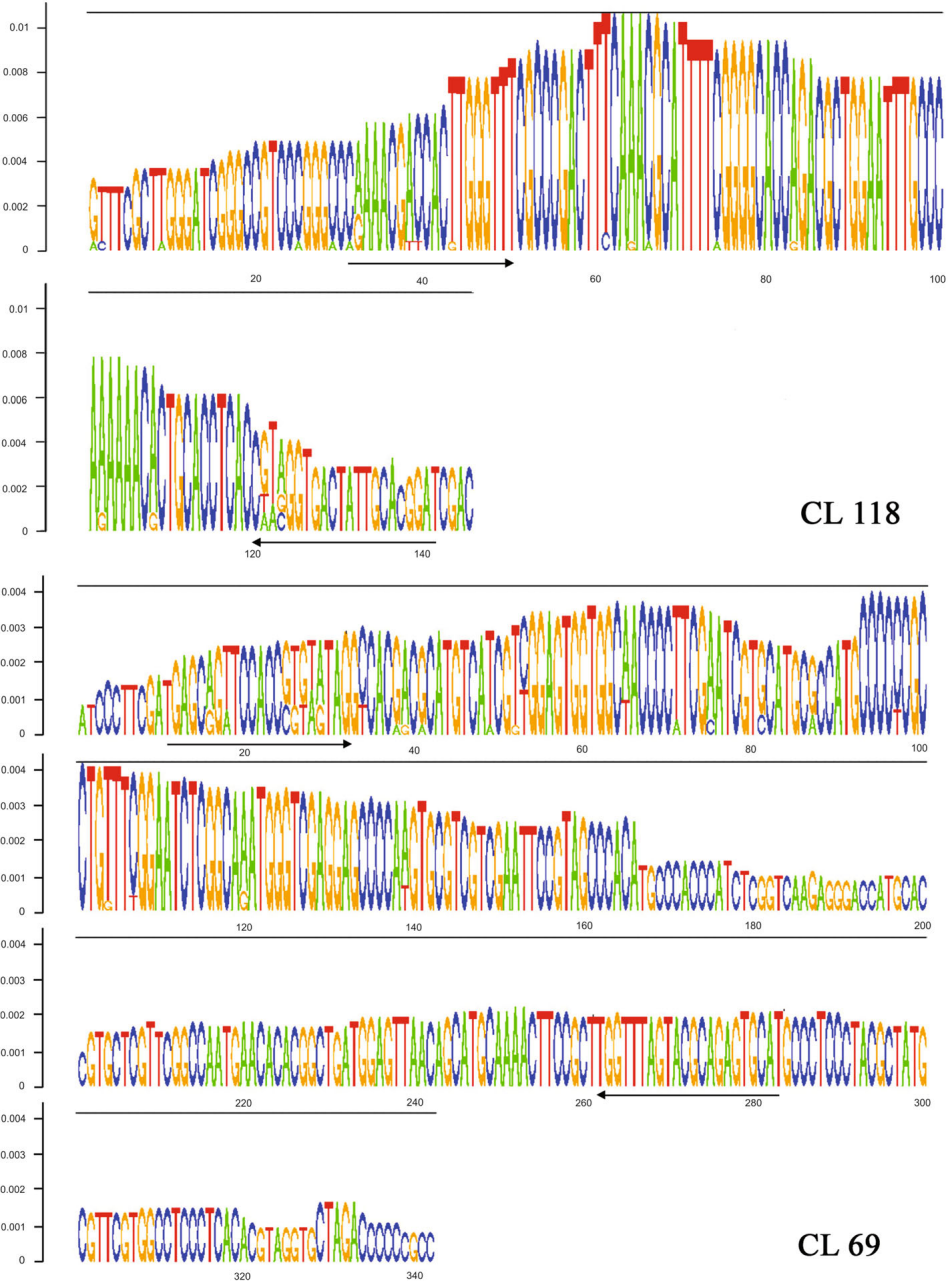
DeBruijn graphs for the consensus sequences of Satellite DNA in *Passiflora edulis*, built from the highest frequency of 5-mers (CL 118–145 pb, PeSat_3) and 13-mer (CL-69 – 342 pb, PeSat_2). The arrows indicate the sense and anti-sense primer sequences used for probe synthesis

Two CLs were identified as Satellite DNAs by TAREAN, named PeSat_3 (CL 118) and PeSat_2 (CL 69), with respective sizes of 145 and 342 pb, and both exhibited graphics with a circular layout. PeSat_3 presented *C* and *P* indexes with values equal to 1.0 and for PeSat_2, the value of *C* was equal to 0.79 and *P* was equal to 0.97 (Table 1). The reads connected in the graph were decomposed into K-mers, 5-mers for CL 118 and 13-mers for CL 69, which refer to all possible substrings (of length k) from the reads of DNA sequencing. The *number of k-mers* and *coverage k-mer* (expected number times of each k-mer is seen) are shown in Table 1. The analysis of k-mers was used for the reconstruction of the monomer and consensus represented by the DeBruijn graph (Fig. 5). Based on the DeBruijn graph, it is possible to select the most preserved sequence for the synthesis of oligonucleotides. However, in the present work, the consensus monomers were used to design sense and anti-sense primers by Primer3 plus program version 4.0. (Fig. 5 and Table 2). The search for local similarities between sequences performed on GenBank/NCBI for both CLs did not identify significant similarities with sequences available in the database. Automatic sorting found no similarities to a potential LTR element or rDNA.

**Table 1:** Results of the TAREAN analysis identifying Clusters (CLs) of Satellite DNA in *Passiflora edulis*

Cluster/Satellite name	Genome proportion (%)	Consensus length (pb)	K-mer number	K-mer coverage	Connected component index (*C*)	Pair completeness index (*P*)	Graph layout
CL118/PeSat_3	0.064	145	5	0.92	1.0	1.0	
CL69/PeSat_2	0.16	342	13	0.76	0.79	0.97

**Table 2:** Characterization of Satellite DNAs and LTR retrotransposons identified in the genome of *Passiflora edulis*

Type analyse	Cluster ID	Reads Size (pb)	Genome Proportion (%)	GC (%)	Classification/Localization	Primers (5′ – 3′)	Number acession GenBank
Based graphics	CL 207 PeSat_1	128	0.01	49	Satellite Colocalised region 5S rDNA	L-ATGCCTCACCCACTGTCTTT R-TGTTTAAGGCGTTTCCTTGC	MF 401643
TAREAN	CL 69 PeSat_2	342	0.16	59	Satellite Colocalised region 45S rDNA	L-TGAGCAGTTCCACCGTGTATAG R-ATGCACTCTGCGTACTAAACCA	MF 401645
	CL 118 PeSat_3	145	0.06	57	Satellite Subterminal	L-CAAAACGACCACTTGGGTTT R-ATCCGTGCAATAGTCACCTACG	MF 401644
Protein domains tools	CL 6	1086	1.32	47	*Ty3/Gypsy/Chromovirus* Pericentromeric regions	L-GGAGCTCCAGTTTTGTTCGT R-TGTCTGCAAAACAGTCCTCAA	MF 401635
	CL 11	411	1.10	47	*Ty3/Gypsy/Chromovirus* Disperse all chromosome	L-ACTGCCGCTCTCTCAGAATC R-TGGCACATTCCGGTTATGTAT	MF 401636
	CL 36	924	0.78	48	*Ty3/Gypsy/Athila* Disperse all chromosome	L-CAGTATGCCTTGTGTTCGAG R-TGCATATGAGTTTGTCCTACG	MF 401639
	CL 43	484	0.67	45	*Ty1/Copia/Angela* Disperse all chromosome	L-TTTCGGCTGAGTTTCAGAAG R-GTGCAGCTCAGTAGGGGATA	MF 401642
	CL 86	1123	0.32	33	*Ty1/Copia/Maximus-Sire* Disperse most chromosome	L-AGCTGTGTTAACGGCTTCAG R-ACTTGGGCATGCTAGTTTTG	MF 401640
	CL 94	2274	0.28	35	*Ty1/Copia/Maximus-Sire* Disperse all chromosome	L-CTTGTTGAACGGCCTAAGGA R-ATTTGGCATCCTCCATCTTG	MF 401637
	CL 135	946	0.11	46	*Ty3/Gypsy/Athila* Disperse most chromosome	L-GCACTTCTCCCAGTTCAGGA R-GGCGGTATGACAGTGGTTCT	MF 401638

**Fig. 6:**
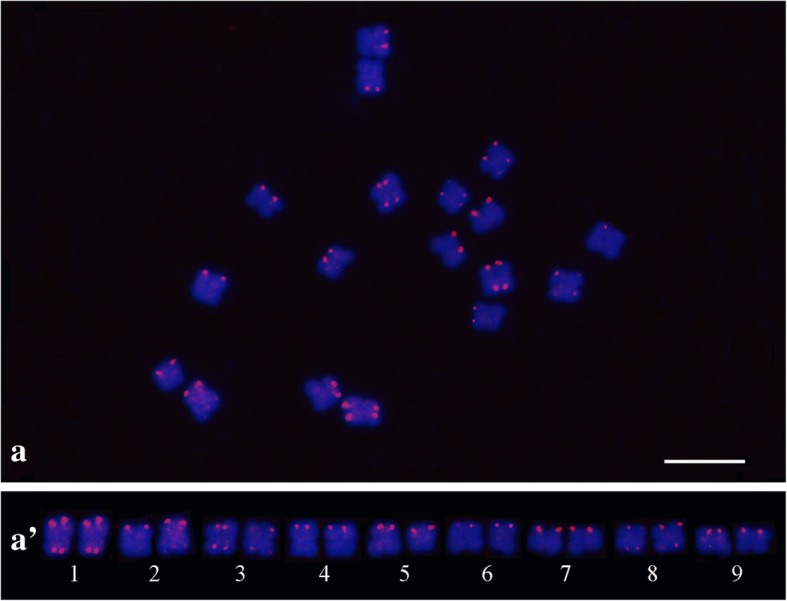
Chromosome mapping by FISH in *Passiflora edulis* (2*n* = 18). Hybridization sites of PeSat_3 (CL 118) (a); karyogram showing three chromosomal pairs with hybridization sites in short and long arms (1, 3 and 8) and in just the short arms of six chromosome pairs (2, 4, 5, 6, 7 and 9) (a’) (Bar = 10 μm)

**Fig. 7:**
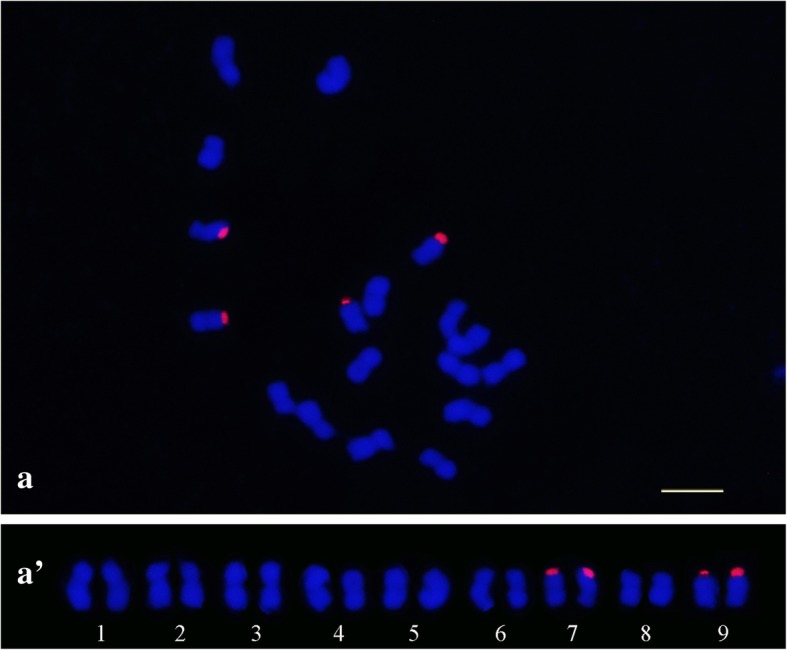
FISH in metaphasic chromosomes of *Passiflora edulis* (2*n* = 18). Hybridization sites of PeSat_2 (CL 69) (a); karyogram showing signals on terminal regions of the short arms of chromosomes 7 and 9 (a’) (Bar = 10 μm)

Chromosome mapping of the PeSat_2 and PeSat_3 satellites revealed distinct hybridization sites, with reproducible and unambiguous markings for all analyzed mitotic metaphases (Figs. 6 and 7). For PeSat_3 the hybridization sites were observed in subterminal regions of the chromosomes, with markings on the short arms and long arms in three pairs of chromosomes (1, 3 and 8), and markings in just the short arms of six chromosome pairs (2, 4, 5, 6, 7 and 9) (Fig. 6a’). Chromosome mapping of PeSat_2 revealed four hybridization sites, with strong signals on terminal regions of the short arms of chromosomes 7 and 9 (Fig. 7).

### Classification and chromosomal mapping of LTR retrotransposons

**Fig. 8:**
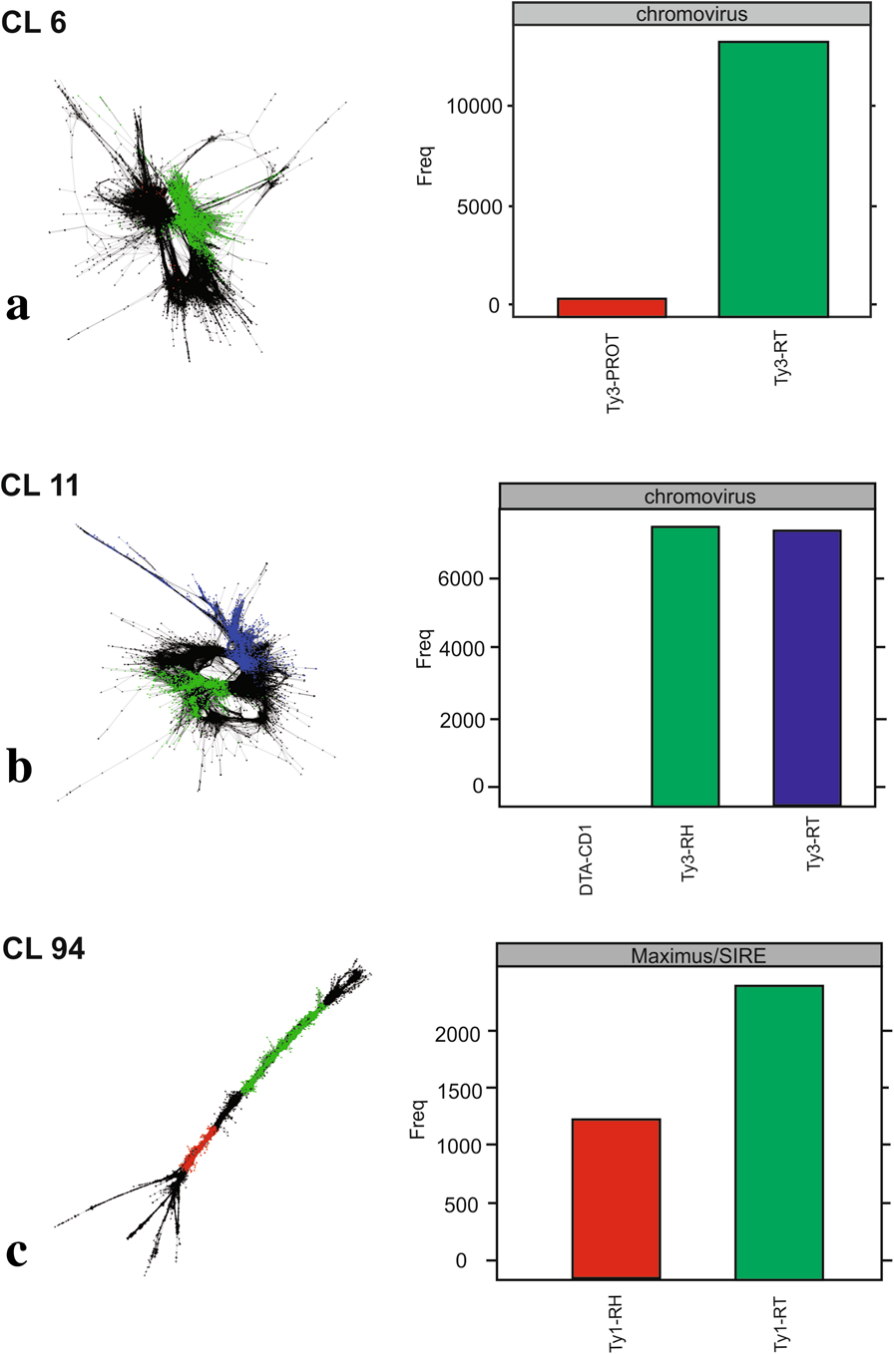
Graphic layouts for CLs 6, 11 and 94 detected by reads clustering with similarities and classified through the RT protein domain in *Passiflora edulis*. CLs 6 (green) and 11 (blue) were classified as Ty3/Gypsy/Chromovirus (**a**, **b**); CL 94 (green) was classified as Ty1/Copy/Maximus-SIRE (**c**)

**Fig. 9:**
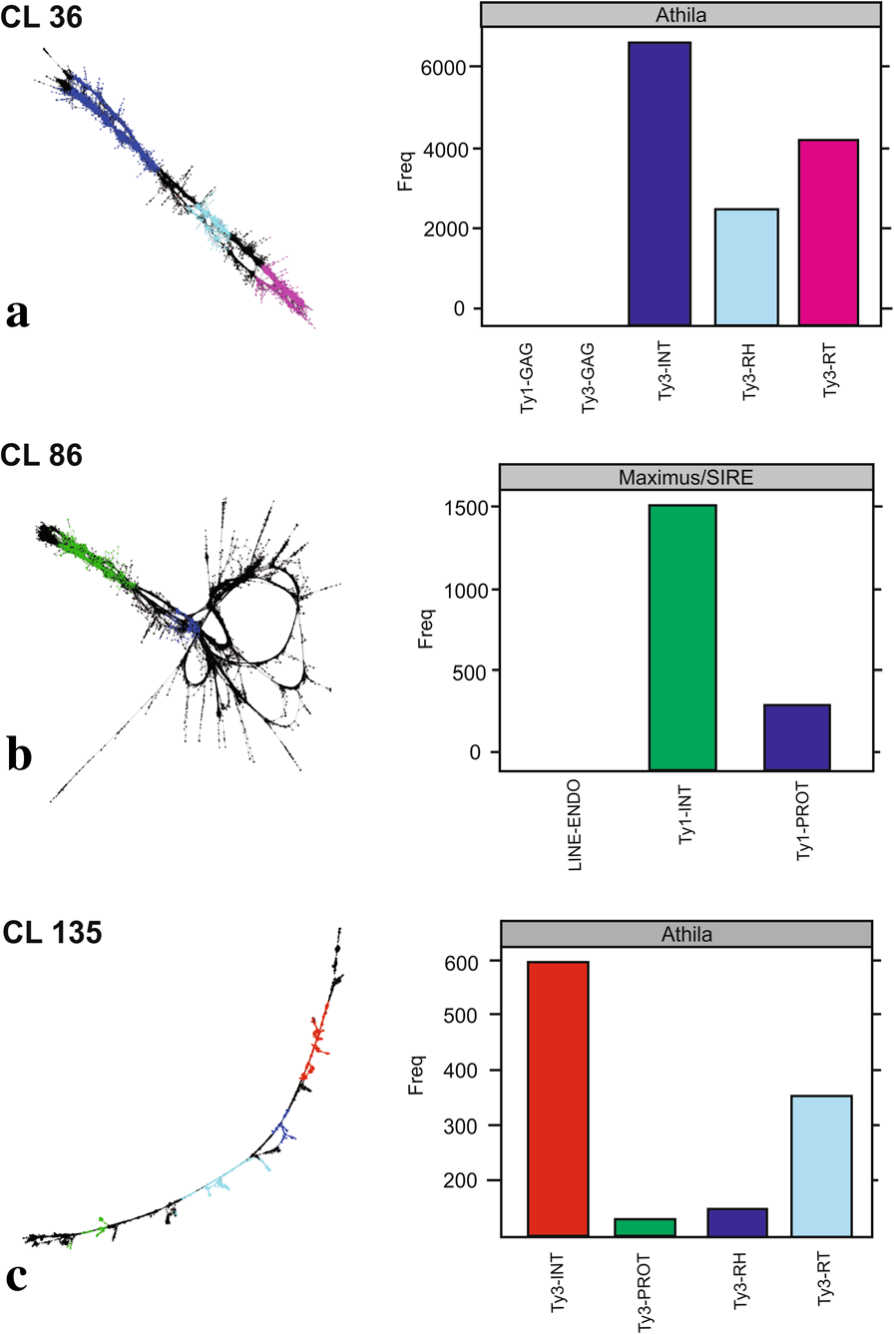
Graphic layouts for CLs 36, 86 and 135 detected by reads clustering with similarities and classified through the INT protein domain in *Passiflora edulis*. CLs 36 (blue) and 135 (red) were classified as Ty3/Gypsy/Athila (**a**, **c**); CL 86 (green) was classified as Ty1/Copy/Maximus-SIRE (**b**)

**Fig. 10:**
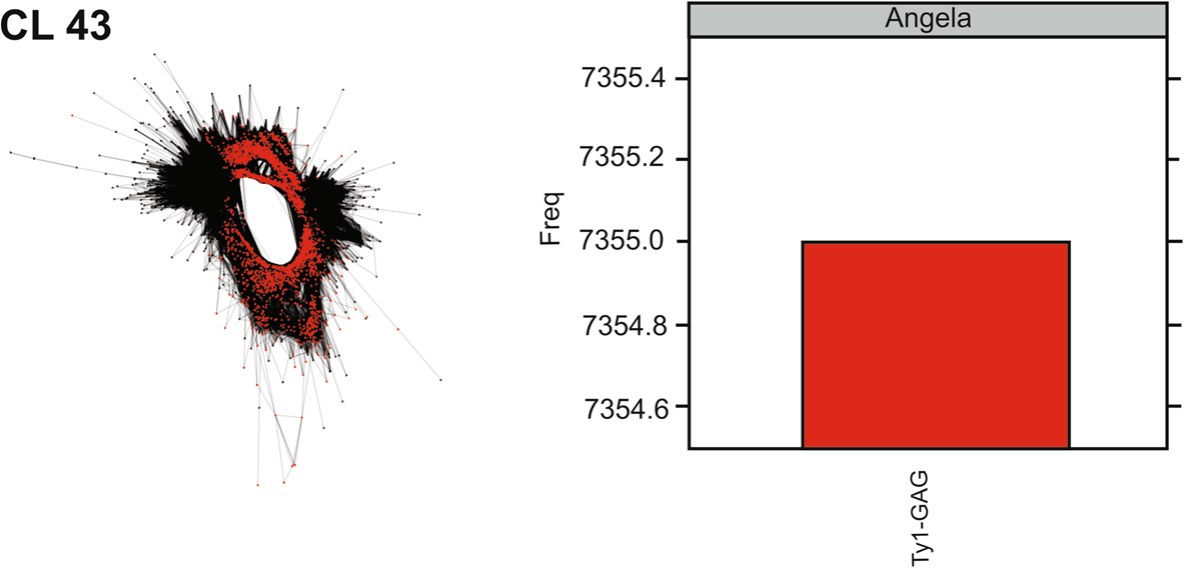
Graphic layout for CL 43 detected by reads clustering with similarities and classified through the GAG protein domain (red) in *Passiflora edulis*, as Ty1/Copy/Angela

The CLs were analyzed regarding their similarity with preserved protein domains RT, INT and GAG of the LTR retrotransposons. Nine CLs were identified with the RT domain, six with the INT domain and eight with the GAG domain. After multiple alignments for the evaluation of similarity between the CLs (data not shown), seven divergent CLs were selected for probes and chromosomal mapping. The selected CLs were 6, 11 and 94 (RT domain), with respective sizes of 1086, 411 and 2274 pb (Table 2); CLs 36, 86 and 135 (INT domain), with respective sizes 924, 1122 and 946 pb (Table 2); and CL 43 (GAG domain), with size of 484 pb (Table 2). Each CL was classified as the superfamily and family for the element. Graphs were built from the grouping of similar reads, where in the domains identified in the CL were represented by different colors. In addition, column graphs show the total numbers of hits similarity for each family, associating the protein domain and the classified element (Figs. 8, 9 and 10).

The preserved RT domain enabled the classification of CLs 6 and 11 as Ty3/Gypsy/Chromovirus (Fig. 8a, b) and CL 94 as Ty1/Copy/Maximus-SIRE (Fig. 8c). For the INT domain, CLs 36 and 135 were classified as Ty3/Gypsy/Athila (Fig. 9a, c) and CL 86 as Ty1/Copy/Maximus-SIRE (Fig. 9b). Finally, for the GAG domain, CL 43 was classified as Ty1/Copy/Angela (Fig. 10).

**Fig. 11:**
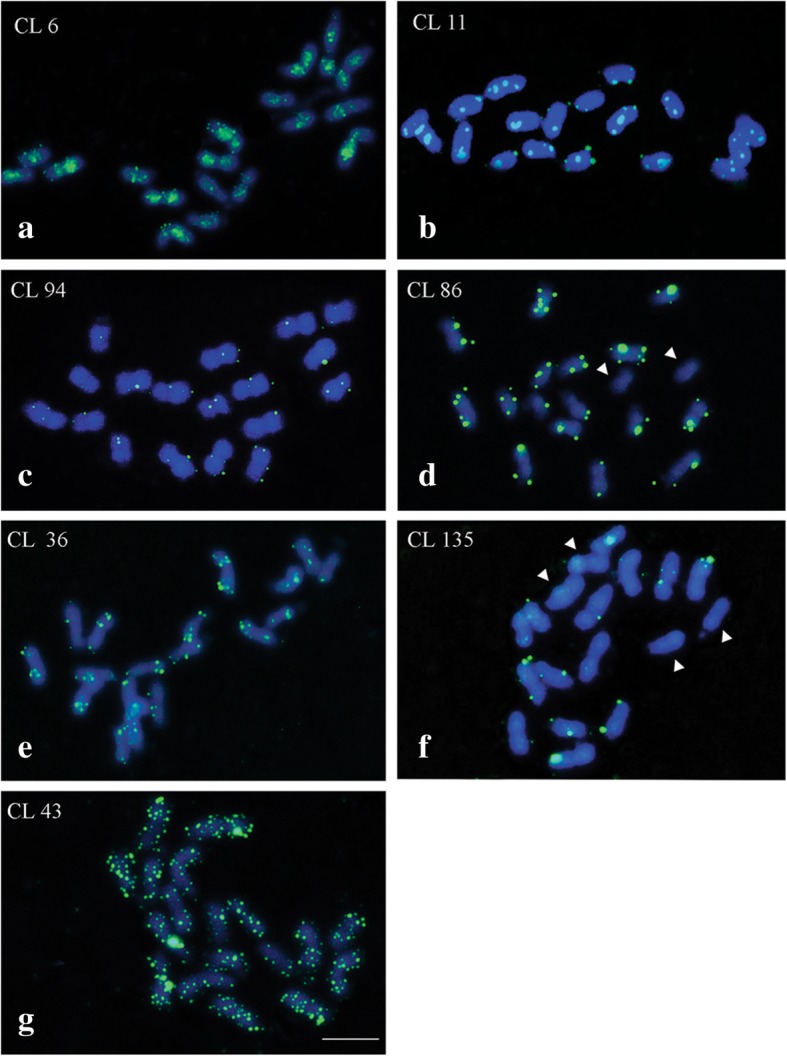
Chromosome mapping of LTR retrotransposons clusters in *Passiflora edulis* (2*n* = 18). Hybridization profiles observed in: CLs 6 and 11 classified as element Ty3/Gypsy/Chromovirus (**a**, **b**); CLs 86 and 94 as element Ty1/Copy/Maximus-SIRE (**c**, **d**); CLs 36 and 135 as element Ty3/Gypsy/Athila (**e**, **f**); CL 43 as element Ty1/Copy/Angela (**g**) (Bar = 10 μm)

**Fig. 12:**
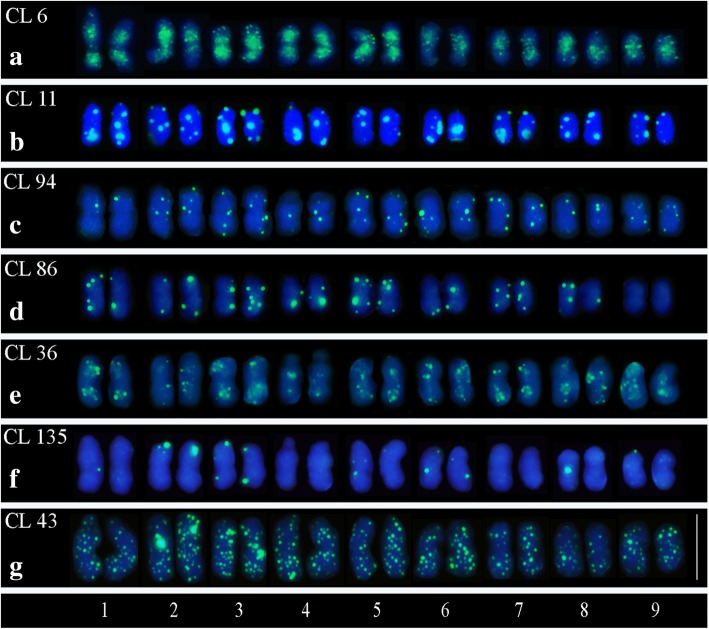
Karyogram for LTR retrotransposons clusters in *Passiflora edulis* (2*n* = 18). CLs 6 and 11 (Ty3/Gypsy/Chromovirus) (**a**, **b**); CLs 86 and 94 (Ty1/Copy/Maximus-SIRE) (**c**, **d**); CLs 36 and 135 (Ty3/Gypsy/Athila) (**e**, **f**); CL 43 (Ty1/Copy/Angela) (**g**) (Bar = 10 μm)

The chromosome mapping for all CLs of protein domains showed distinct and reproducible markings on the analyzed mitotic metaphases, with scattered patterns in most chromosomes. The distribution pattern was similar among most retrotransposons. CL 6 hybridized mainly in the pericentromeric regions, showing signals in the interstitial regions, which diminish or disappear in centromeric and telomeric regions (Figs. 11 and 12a). CLs 11, 36, 94, 86 and 135 presented strong hybridization signals, with specific and dispersed sites in most chromosomes (Figs. 11 and 12b, c, d, f). CLs 86 and 135 were the only ones without hybridization sites in some of the chromosome pairs. More specifically, CL 86 did not present hybridization sites in the last chromosome pair and CL 135, in the fourth and seventh chromosome pairs (Figs. 11 and 12d, f). CL 43 presented the greatest abundance and distribution of hybridization sites in all chromosomes, with very strong signals (Figs. 11 and 12g).

## Discussion

The in silico genomics analysis made it possible to characterize repetitive DNA sequences in *P. edulis*, as well to determine their in situ location in the karyotype by FISH. The RepeatExplorer pipeline, used for in silico analysis, has been widely used in the study of repetitive DNA in plants, and publications are increasing. The RepeatExplorer has many advantages because it does not require a reference genome for contigs assembling, offering an easy-to-use interface, free webserver, pre-processing of the sequencing data, fast analysis and with detailed and robust results.

In *P. edulis,* the low-coverage sequencing (2.2x) was enough to provide a good representation of the repetitive sequences. A very similar proportion was obtained for *Coccicinea grandis*, with 2.7x genomic coverage, providing satisfactory results in the analysis of TEs by RepeatExplorer [44]. Other works have shown the efficiency of low-coverage sequencing in studies with repetitive DNA [42, 45–48].

The graph-based clustering of reads has identified a high proportion of repetitive DNA in the genome of *P. edulis*, around 59% (Fig. 2). The high value of repetitive DNA is commonly found in plant genomes [8]. Among the classified types of repetitive DNA, there was a higher prevalence of LTR retrotransposons, amounting to 53% (Fig. 2). TEs (DNA transposons or retrotransposons) generate substantial variation in genome size in several species when performing their own drive mechanisms, such as observed in maize, in which TEs represent 90% of the genome [49]. LTR retrotransposons are the most abundant elements in the genome of plants, possibly because they perform their transposition mediated by mRNA through a replication mechanism, known as “copy and paste”, thus increasing the size of the genome [18, 20]. The long repetitive sequences present at the endings of the retrotransposons do not encode polypeptide but play an important role in the regulation of retroelements [21].

The LTRs classification revealed a frequency twice as high for superfamily Ty3/Gypsy (33.33%) in the genome when compared to Ty1/Copy (16.89%) (Fig. 2). The genomes for *Pisum sativum* L., *Glycine max, Silene latifolia* Poir., *Feestuca pratensis* Huds., *Solanum lycopersicum* and three Helianthus species have also shown higher prevalence of Ty3/Gypsy over Ty1/Copy [43, 47, 50–52]. However, in the Eleocharis genome was found greater predominance of Ty1/Copy compared to Ty3/Gypsy [53]. The main difference between superfamilies Ty3/Gypsy and Ty1/Copy is in the structural organization of their intermediate polyprotein molecule, and both are the most frequent and diversified forms in the genomes of eukaryotes [16, 21].

Contrary to the abundance of retroelements, studies have reported low frequency of Satellite DNAs in plant genomes. In the present study, only two among the 223 CLs obtained were classified as satellites, representing less than 0.1% of the genome (Fig. 2). Corroborating this result, genomic analyses of *Passiflora* through the BAC-end library sequencing also showed a very low number of SatDNAs; only one was characterized as satellite among 4774 repetitive elements founded [31]. Similarly, after examination of *Coccinia grandis* L. Voigt by RepeatExplorer, only two of the 21 repetitive elements were satellites [44]. In turn, 3% of the genome was classified as satellite in *Glycine max* L., which is considered high for this species [43]. The low proportion of SatDNAs, commonly found in in silico analyses, may be related to the high variability among and between species, which makes it difficult the identification of sequences with homology [44].

Satellite DNAs are composed by highly repetitive sequences *in tandem*, and are generally located in heterochromatin regions (found mainly in centromeric or subtelomeric regions) [8, 21]. The SatDNA hybridization of PeSat_1 (CL 207) was expected in the centromeric region, due to its characterization as SatDNA, circular graph and hits with similarities to centromere DNA in other species. However, the presented profile was not centromeric, with hybridization sites restricted to a pair of homologous chromosomes co-located with the 5S rDNA sites (Fig. 4). As rDNA presents many copies *in tandem*, it justifies the circular layout of the graph. Because two contigs of CL 207 (not used for the synthesis of the probe) showed homology to 5S ribosomal gene through GenBank, this hybridization result can be justified by the proximity of the reads grouped in the same CL, even when homology has not been verified in the databases for that contig. In addition, the 5S rDNA consists of repetitions units containing a transcription region with approximately 120 pb, and non-transcribed spacer (NTS) with highly variable size (100–700 pb). Because the coding region is highly preserved and the NTS region varies widely between species, the variance between genus can be due to divergence of the NTS sequence [54]. Thus, it is presumed that the sequence determined by CL 207 (128 pb) is a satellite associated with the NTS region. The polymorphisms of the NTS regions (size variation and chromosome distribution) can be used to compare species from different clades and suggest evolutionary mechanisms. 5S rDNA non-transcribed spacers (5S-NTS) sequences have being used as phylogenetic markers in plant species, as cotton [55], wheat [56], goosefoot [57], and orchid [58], among others.

In the present study was not possible to identify the centromere DNA for *P. edulis*, indicating a highly species-specific, low-preserved centromeric DNA, considering that it was not observed significant homology with other plant species for precise classification. Centromeres can also present a large number of retrotransposons or a single-copy DNA, thus interfering in the detection of *in tandem* repeats [22, 59, 60]. Centromeres formed mainly of single-copy DNA has been observed in five potato centromeres, and no satellite repetition has been identified [61]. Ten distinct families of centromeric retrotransposons were grouped in the genus Coffea [60] and Sugarcane centromeres contain both satellite and retrotransposon DNAs [62].

The tools used in this work did not enable the identification of isolated centromere DNA sequences. Therefore, other strategies can be employed for *P. edulis* based on the isolation of DNA in this region, as performed by *Chromatin immunoprecipitation* (Chip), which uses an antibody against a specific protein in the centromere/kinetochore complex for immunoprecipitation, so that the linked DNA co-precipitates and can then be sequenced (Chip-seq) [25, 62, 63].

TAREAN is a tool that uses k-mers frequency, which is more suitable for the reconstruction of monomers from a non-assembled short-sequence reads, and therefore can complete the gaps unfilled by graph-based clustering [46]. In *P. edulis*, TAREAN enabled the detection of two new satellites repeats, undetected on the previous analysis. PeSat_3 (CL 118) was characterized as *high-confidence satellite* for presenting *C* and *P* indexes equal to one, and PeSat_2 (CL 69) was characterized as *low-confidence satellite* (*C* = 0.79, *P* = 0.97), respecting the recommended values of *C* > 0.7 and *P* > 0.4 for this classification (Table 1). *Vicia faba* L. was the species with the highest number of satellites registered using TAREAN, with 11 new identified satellites [46].

The chromosomal hybridization observed for PeSat_3 (145pb) has shown signals at the subtelomeric location (Fig. 6). Similar result was observed in *S. latifolia*, in which a 159 pb satellite was mapped in the subtelomeric region of all chromosomes, and in both arms of most chromosomes [47]. Subtelomeric satellites have been identified in many other plant species, such as potatoes [64], rice [65] and maize [66]. In *P. edulis*, a very specific pattern was observed for this satellite, present in both arms of three chromosomes and only in the short arms of six chromosomes (Fig. 6). SatDNA sequences are generally species/genus-specific [8], and can help in comparative studies to a better understanding of the evolutionary history of *Passiflora*.

The results achieved by FISH for PeSat_2 show its co-located with 45S rDNA sites (Fig. 7), presenting four hybridization sites in the same locus for the 26S rDNA probe (data not shown). The 342pb size of the consensus monomer may indicate that these sequences are derived from the IGS region (large intergenic spacers). In eukaryotes, the general standard of organization of rDNA genes is similar, wherein each unit is formed by rDNA genes (18S, 5.8S and 26S), which are separated from one another by small internal transcribed spacers (ITS), and the gene units are separated by large intergenic spacers (IGS), composed by small repetitive sequences (100 - 300pb), that are not transcribed but functional in the regulation of genes [67]. In *S. latifolia*, after analysis of the graph and reconstruction of the sequences, a SatDNA with 313pb was identified, derived from the IGS region of the 45S rDNA [47]. The nearly circular format of the graph for PeSat_2 (Table 1) reflects the *in tandem* organization of the multiple copies of the rDNA repeat unit, and were therefore detected as *low-confidence satellites* by Tarean. In *V. faba*, a consensus monomer with 168pb, characterized by TAREAN as *low-confidence repetition satellites*, also presented satellites derived from the IGS region of the 45S rDNA [46]. The spacer sequences do not contribute to the rRNA synthesis, are under low selective pressure, and are susceptible to high rates of mutation, and therefore are not preserved between species but within species. These features allow the use of spacer regions as a molecular tool for classification at species level and can recognize recent changes in evolutionary history [67].

LTR retrotransposons comprise a group of repetitive DNAs in the genome of plants, with sequence sizes varying between 300 to 5000 nucleotides [21]. The CLs of *P. edulis*, classified as LTRs, have not represented the entire element, and the sizes observed ranged from 411 to 2274 nucleotides (Table 2). The graph layouts obtained in the analyses of protein domains revealed a variation between circular, linear and circular/linear (Figs. 8, 9 and 10). The circular layout is characteristic to either SatDNA or terminal regions repeats, such as LTR retrotransposons. These linear graphs result from a lack of sufficient coverage of the sequencing or from the presence of low-coverage variable sequence regions [43]. The sequences must be sufficiently frequent in the genome to be represented in low-coverage data, must be identified as repetitions and quantified with precision [47].

A directly proportional relationship between abundance of CL in the genome and hybridization signals has been observed, i.e. the higher the proportion in the genome, the greater the number of scattered sites in the chromosomes. CL 6 presented higher proportion in the genome (1.32%) and CL 135 presented the lowest (0.11%) (Table 2). Thus, more hybridization sites have been observed for CL 6 than for CL 135 hybridizations (Figs. 11e and 12). However, in the sequences mapping was observed that the hybridization signals were greater for CL 43 (0.67%) than for CLs 11 and 36 (1.10and 0.78%, respectively), which may be related to an underestimation of the proportion in the genome for CL43 (Fig. 12).

The repetitive DNA concerning to TEs can appear scattered in the genome, or restricted to specific locations when *in tandem* repeats [8]. In *P. edulis*, the LTRs are either scattered or grouped in the chromosomes. In *C. grandis* CL 10, classified as Ty1/Copy/Angela presented weakly scattered signals in the chromosomes, and CLs 9 and 37, classified as Ty3/Gypsy/Athila, showed signals grouped in the subterminal or the pericentromeric region [44]. Unlike other CLs with scattered hybridization sites, only the CL 6 (Ty3/Gypsy/Chromovirus) presented preferential association sites in the pericentromeric regions in *P. edulis* (Figs. 11e and 12a). Comparing the hybridization signals for *C. grandis* and *P. edulis,* the Ty3/Gypsy elements were observed grouped, and the Ty1/Copia elements always showed signals scattered. However, more detailed studies are needed to understand and validate these observations.

The CLs 6 and 11, both classified as Ty3/Gypsy/Chromovirus, showed different patterns of chromosome hybridization sites, wherein CL 11 did not present preferred sites, showing a diversification of this family in the genome (Figs.11e and 12a, b). The chromovirus have a chromodomain with a role in chromatin remodeling and in the regulation of gene expression during the development of eukaryotes [68]. This retroelement can be associated with an important regulatory function of histone-modifier enzymes and the maintenance of pericentromeric heterochromatin, which participates in the control of recombinations involving the centromere [69, 70].

Four TEs families have been identified between the superfamilies Ty1/Copy and Ty3/Gypsy. The families Chromovirus (CLs 6 and 11), Maximus-Sire (CLs 86 and 94) and Athila (CLs 36 and 135) were each represented in two CLs and the Angela family was represented only in CL 43 (Table 2). Recent analysis for *Hippophae rhamnoides* L. showed that the most families of TEs were represented by one or two clusters, and few were found in multiple clusters, suggesting that when the families are represented in few clusters, they are called conserved families, presenting no changes in the sequence and structure of the element [71]. In turn, families that are present in several clusters indicate high divergence. Thus, we can suggest that *P. edulis* presents a conserved pattern for LTR retrotransposon families.

TEs represent the widest diversity among genomes of phylogenetically similar organisms [20]. TEs are considered sources of new genetic and regulatory information of the genome, and may influence the expression and the dynamics of genetic information, thereby acting directly in the genomes evolution [21]. Studies of diversity, organization and distribution of TEs are important to understanding the role of these elements in the genome.

## Conclusions

New information was generated about the repetitive DNA of the *Passiflora edulis* genome based on NGS data. The high proportion of repetitive DNA identified by low-coverage sequencing reflects in a higher proportion of LTR retrotransposons of the Ty3/Gypsy superfamily, and these are one of the main responsible elements for the species genome size. The analyses of LTR retrotransposons have contributed to a better understanding of the genomic organization of the TEs in *P. edulis* mostly presenting scattering patterns, and a single pericentromeric marking element, all with plenty of relevant differences in the genome. A few SatDNAs have been observed, with two of them associated to the rDNA regions and one of them to the subtelomeric region, acting as a cytological marker for chromosome organization, considering that those sequences are usually species/genus-specific. Therefore, the information generated in this work provides a starting point for further investigations of *Passiflora* genome; besides comparisons to related species, which could help the cytogenomic comparison and the the understanding of evolutionary patterns of repetitive sequences and their impact on other scientific areas as toxonomy, phylogeny and breeding.

## Methods

### Plant material and cytological preparation

Samples of *Passiflora edulis* (2*n* = 18) were collected from commercial populations of passion fruit-producing farms in the municipality of Livramento de Nossa Senhora, the state of Bahia (BA), Brazil (latitudes 13°17′ and 15°20′ S and longitudes 41°05′ and 43°36′ W). The plants were kept at the Germplasm Active Bank (BAG-Passifloras), located at the State University of Santa Cruz (UESC), in the city of Ilhéus, the state of Bahia, Brazil (latitude 14°39′ S, longitude 39°10′ W, altitude 78 m). Stakes were arranged in bags with sand, and after 15 days the roots were collected with about one centimeter in length. The root tips were pre-treated in 8-hydroxyquinoline solution (8-HQ) at 0.0 02 M at room temperature (RT) for 1 h and an additional 21 h ± 8 at 10 °C, then washed twice for 5 min in distilled water, fixed in Carnoy I (ethanol/acetic acid, 3:1, *v*/v; [72]) for 3 h at RT, then stored at − 20 °C for at least 24 h or until use. The samples were washed twice for 5 min and incubated in enzymatic solution at 2% cellulase and pectinase at 20% for 80 min at 37 °C. After enzymatic digestion, the rootlets were washed with distilled water and dried with filter paper, then 6 μL of 60% acetic acid was added and they were macerated with the help of a needle and stereoscopic microscope, covered with cover slips, pressed gently with filter paper and frozen in liquid nitrogen for at least 5 min. The coverslips were removed with a scalpel, air-dried and stored at − 20 °C until the selection of slides with good metaphasic cells to carry out the FISH.

### Genomic DNA extraction

The genomic DNA extraction was performed according to the protocol described by Doyle and Doyle [73], with modifications for *Passiflora* [74]. The genomic DNA was purified with the addition of 10% sodium acetate (3 M, pH 5.2) and 200% of the final volume of the anhydrous ethanol at − 20 °C. The quantification of the extracted DNA was performed on Qubit 2.0 fluorometer (Termo Fisher Scientific), using the Qubit dsDNA kit (Q32850). The samples quality was checked by the absorbance ratio 260/230 and 260/280 in Nanodrop equipment (Termo Fisher Scientific).

### Next-generation sequencing (NGS)

The genomic library was built using the Nextera DNA Sample Preparation kit (Illumina®) with the Nextera index kit (Illumina®), strictly following the manufacturer’s recommendations. Firstly, the fragmentation was performed with 50 ng of the genomic DNA, with purification using the Illustra GFX PCR DNA and the Gel Band Purification kits (GE Healthcare Life Sciences); amplification and linkage of the indexes (72 °C for 3 min, 98 °C for 30 s, 5 cycles of 98 °C for 10 s, 63 °C for 30 s, and 72 °C for 3 min), and purification was performed by magnetic beads (AMPure XP beads GEHelthcare Life Sciences) and washes with 80% ethanol. The genomic library was quantified with KAPA Library Quantification Kit Illumina®Platforms (KR0405), in ABI Prism real-time PCR equipment (Applied Biosystems), following the manufacturer’s protocol for the preparation of the qPCR reactions. The qualitative assessment of the libraries was inferred by the dissociation curve analysis of the graph obtained after qPCR, wherein the presence of adapter dimers was also evaluated. The sequencing was performed at the Laboratory of Molecular Markers at the Center of Biotechnology and Genetics (CBG), UESC, Bahia, Brazil, using the Illumina MiSeq® platform with the MiSeq® reagents kit V3 600 cycles (Illumina®). The methodology strictly followed the “MiSeq® Reagent Preparation Guide (catalog number 15.044.983)”, as well as the “MiSeq® System User’s Guide (part no. 15.027.617_PTB)”.

### Bioinformatics using RepeatExplorer

The identification and characterization of the repetitive DNA families was performed using the RepeatExplorer pipeline [42, 43], implemented in the Galaxy server (http://repeatexplorer.org/), which uses NGS reads for analysis. A total of 11,493,782 paired-end sequence reads (average size of reads 300 pb) was obtained by sequencing, with 43% of GC content and genomic coverage of 2.2x (1C = 1.545.24 Mpb, [75]). The formula used to calculate the genomic coverage was Cov = (N x L)/G, wherein N represents the number of paired-end reads, used in the analysis, L represents the size of reads and G is the size of 1C content of the species’ genome. The adapters were removed with a tool available on the Illumina® platform, and the quality control of the sequencing data were accessed by FastQC (version 0.11.4).

#### Graph-based identification of repetitive DNA

Cluster analysis was performed using a graph-based approach to identify clustered read repeats de novo, without the need for a reference genome [41]. Initially, was performed the preprocessing of the reads. The reads were filtered in terms of quality using a cut-off of 30, trimmed and filtered by size (100 pb) to obtain high-quality reads. Interlaced paired reads were sampled randomly to cover 5% of the genome of the species (772,620 reads). The number of analyzed reads represented 0.15x of the coverage of the genome (recommended value ranges between 0.01–0.50x). Clustering of the reads was accomplished with a minimum overlap of 55 and 90% similarity. In addition to the characterization of clusters (CLs) using the RepeatMasker database, now available in the program, a custom database was built with consensus repetitive sequences for centromeric regions and TEs associated with the centromeric region. This database was constructed from public databases (Repbase, most commonly used database for repetitive DNA, Plant Repeat Database^1^ and NCBI-National Center for Biotechnology Information) totaling 11,868 sequences. At the end of the analysis by RepeatExplorer, the probable CLs containing Satellite DNAs were selected from the automatic classification and the graphic layout. The contigs with highest abundance index in the CL were used for prime design and probe preparations. The sequences were included in the GenBank (Table 2).

#### Identification of satellite DNA by the tandem repeat analyzer (TAREAN)

The TAREAN tool available in RepeatExplorer [46] was used for the identification of Satellite DNA. The TAREAN is based on the analysis of reads graphs for the identification of clustered satellites. Later, it used k-mers frequency statistics in the reconstruction of molecular consensus for each satellite CL. The automatic detection of repetition satellites was based on the parameters “*Connected component index* (*C*)” and “*Pair completeness index* (*P*)”. These are characterized as *high-confidence satellites* when both assume values close to one. Parameters *C* > 0.7 and *P* > 0.4 are characterized as *low-confidence satellites*. The analyses were performed with 250,000 input reads and CL merging option. Both *low* and *high confidence satellites* CLs were used for the design of primers and probes for FISH. The identified monomers had their sequences included in the GenBank database (Table 2).

#### Identification of protein domains of LTR retrotransposons

The *Protein Domain Search* tool [42] was used for the identification and analysis of protein domains of LTR retrotransposons, using selected clusters (CLs) analysis as input file. The tool performed analysis of sequences similarity of *Passiflora* with a database of protein domains for RT, INT and GAG. The output generated for each domain was subjected to filtering with stringency parameters (minimum of 60% similarity and 40% identity). The information contained in the reference sequences for protein domains allowed the definition of the superfamily level (Ty1/Copy and Ty3/Gypsy) and the family level (Athila, Angela, Chromovirus, Maximus-SIRE, among others). Among the CLs identified in this analysis, the sequences similarities were verified through multiple alignments using MUSCLE on the Phylogeny.fr platform (v 3.8.31) [76, 77]. Among the CLs identified in this analysis, the most divergent CLs were used for chromosomal mapping and their sequences were included in the GenBank (Table 2).

### Production of probes for FISH

The primers for amplification of Satellite DNAs and retroelements protein domains were designed in Primer3Plus [78]. The amplification reactions were prepared on a volume of 50 μl containing 10 ng/ul of gDNA of *P. edulis*, 1 mM dNTPs, PCR buffer 10X, 50 mM MgCl_2_, 10 μM of each primer, 1 U Taq polymerase (Vivantis) and ultrapure water to complete the desired volume. The PCR was performed in thermocycler (Eppendorf Mastercycler,) using the program: 4 min at 94 °C for initial denaturation, followed by 30 cycles of 1 min at 94 °C, 1 min at 56 °C and 2 min at 72 °C. At the end, there was an additional 10-min extension at 72 °C. In order to verify that the expected fragment amplification occurred, PCR products were subjected to electrophoresis in 1% agarose gel using DNA molecular weight marker (50pb) (Invitrogen™ Life Tecnologies). The gel image was captured under ultraviolet light by photo-documentation device L-Pix (Loccus Biotecnologia).

The Satellite DNA probes were marked with digoxigenin-11-dUTP via Nick Translation Mix (Roche, 11,209,256,910), with a final concentration of 1 μg of the purified PCR product, following the protocol proposed by the manufacturer. The retroelements probes were marked via PCR with biotin-16-dUTP (Roche, 11,093,070,910), through a re-PCR of the purified PCR product with the following dNTPs concentration: 1 mM dATP, dCTP and dGTP, 0.65 mM dTTP and 0.35 mM biotin-16-dUTP.

### Fluorescent in situ hybridization (FISH)

The slides treatment for FISH followed the protocol proposed by Schwarzacher and Heslop-Harrison; and Souza et al. [79, 80], with modifications made by Melo et al. [81]. Cytological preparations and selected slides with good metaphases were dried in an oven at 37 °C for 1 h. The slides were treated with 50 μg/mL RNase in 2xSSC buffer (0 .3M sodium chloride; 0. 03 M sodium citrate) and incubated in humid chamber for 1 h at 37 °C. The slides were then immersed in 2xSSC twice at RT for 5 min, treated with 50 μL of 10 mM HCl for 5 min, then added 50 μL of 10 mg/mL pepsin solution and 10 mM HCl (1:100 *v*/v), then the slides were incubated in humid chamber for 20 min at 37 °C. Later, the slides were washed in 2xSSC twice at room temperature for 5 min, immersed in 4% formaldehyde at room temperature for 10 min, and washed twice in 2xSSC for 5 min. The dehydration step was performed in 70% ethanol and 96% ethanol, 5 min each. After drying the slides at room temperature for 30 min, the hybridization mix with final volume of 15 μl was added, containing 50% formamide, 10% dextran sulphate, 2xSSC (salt, sodium citrate; Sigma), 0.13% sodium dodecyl sulphate (Bioagency) and 50 ng of DNA probe. The hybridization mix was heated to 75 °C for 10 min in thermocycler (Eppendorf, Mastercycler) and immediately transferred to ice for 5 min. The slides containing the hybridization mix were denatured in thermocycler (Techne, TC-412), containing a slide adapter, at 75 °C for 10 min and incubated overnight in humid chamber at 37 °C. After hybridization, the slides were immersed in 2xSSC at room temperature for 5 min to remove the cover slips. The slides were incubated in water bath (Marconi, MA093/1/E) at 42 °C, in 2xSSC twice for 5 min, in 0.1xSSC twice for 5 min, and in 2xSSC twice for 5 min. The slides were immersed in solution with 0.2% 4xSSC/Tween 20 (Sigma) for 5 min at room temperature, and treated with 50 μl of 5% bovine serum albumin, fraction V (BSA; Sigma). The probes marked with biotin-16-dUTP were detected with 0 .7μl avidina-fluorescein isothiocyanate (FITC-Avidin; Vector) plus 19.3μl of 5% BSA per slide. The probes marked with digoxigenin-11-dUTP were detected with 0 .7μl anti-digoxigenin-rhodamine (Roche) plus 19.3μl of 5% BSA per slide. The slides containing the antibodies for detection were incubated in humid chamber for 1 h at 37 °C. To remove the antibody excess were performed three 5-min rinses with 0.2% 4xSSC/Tween20 at room temperature. The slides were briefly immersed in 2xSSC and simultaneously assembled and counter-stained with Vectashield Antifade Mounting Medium with DAPI (H-1200). The slides were then stored at 8–10 °C until analysis.

### FISH analysis and photo-documentation

The hybridization analysis and the photo-documentation were performed with the use of an epifluorescence microscope Olympus BX41 equipped with 5MP digital camera Olympus DP25 and DP2-BSW software. DAPI was visualized with U-MWU filter (330-385 nm excitation / 400 nm dichroic cut-off / emission > 420 nm). The hybridizations detected with avidin-FITC were visualized with the U-MWB filter (450-480 nm excitation / 500 nm dichroic cut-off / emission > 515 nm) and the hybridizations detected with anti-digoxigenin-rhodamine were visualized with the U-MWG filter (510-550 nm excitation / 570 nm dichroic cut-off / emission > 590 nm). The overlaps of Rhodamine/DAPI for satellites and FITC/DAPI for retroelements were performed with the use of Photoshop SC5 software.

## Abbreviations

BA: Bahia State
BAG: Germplasm Active Bank
*C*: Connected component index
CBG: Center of Biotechnology and Genetics
Chip: Chromatin immunoprecipitation
Chip-seq: Chip sequencing
CLs: Clusters
FISH: Fluorescent In Situ Hybridizations
gag genes: Group-specific antigen
IBGE: Brazilian Institute of Geography and Statistics
IGS: Large intergenic spacers
INT: Protein integrase
ITS: Internal transcribed spacers
LTR: Long Terminal Repeat
NCBI: National Center for Biotechnology Information
NGS: Next-Generation Sequencing
NTS: Non-transcribed spacer
*P*: Pair completeness index
PeSat: Satellite DNA *Passiflora edulis*
pol genes: Polyprotein
rDNA: ribosomal DNA
RT: Reverse transcriptase
SatDNA: Satellite DNA
TAREAN: Tandem Repeat Analyzer
TEs: Transposable elements
UESC: State University of Santa Cruz

## Footnotes

1
*The site of the plant repeat database was decommissioned on February 8, 2017 due to the lack of sufficient funding in February 2017.*
