# Correction to: low coverage sequencing for repetitive DNA analysis in *Passiflora edulis* Sims: citogenomic characterization of transposable elements and satellite DNA

**DOI:** 10.1186/s12864-019-5678-1

**Published:** 2019-04-18

**Authors:** Vanessa Carvalho Cayres Pamponét, Margarete Magalhães Souza, Gonçalo Santos Silva, Fabienne Micheli, Cláusio Antônio Ferreira de Melo, Sarah Gomes de Oliveira, Eduardo Almeida Costa, Ronan Xavier Corrêa

**Affiliations:** 1 Departamento de Ciências Biológicas, Universidade Estadual de Santa Cruz (UESC), km 16, Salobrinho, Ilhéus, Bahia CEP 45662-900 Brazil; 2 CIRAD, UMR AGAP, F-34398 Montpellier, France; 3 Departamento de Botânica, Instituto de Biociências, Universidade de São Paulo (USP), Rua do Matão, 14 – Butantã, São Paulo, SP CEP 05508-090 Brazil; 4 Núcleo de Biologia Computacional e Gestão de Informações Biotecnológicas (NBCGIB), Universidade Estadual de Santa Cruz (UESC), km 16, Salobrinho, Ilhéus, Bahia CEP 45662-900 Brazil

**Correction to: BMC Genomics (2019) 20:262**

**https://doi.org/10.1186/s12864-019-5576-6**

Following publication of the original article [1], the authors reported that one of the authors’ names was typeset incorrectly. In this Correction the incorrect and correct author name are shown. The original publication of this article has been corrected.

Originally the author name was published as:

- Pamponét Vanessa Carvalho Cayres

The correct author name is:

- Vanessa Carvalho Cayres Pamponét
